# Replicative Senescence in Mesenchymal Stem Cells: An In Vitro Study on Mitochondrial Dynamics and Metabolic Alterations

**DOI:** 10.3390/antiox14040446

**Published:** 2025-04-08

**Authors:** Beatrice Casorati, Isabella Zafferri, Sara Castiglioni, Jeanette A. Maier

**Affiliations:** Department of Biomedical and Clinical Sciences, Università di Milano, 20157 Milano, Italy; beatrice.casorati@unimi.it (B.C.); isabella.zafferri@unimi.it (I.Z.); jeanette.maier@unimi.it (J.A.M.)

**Keywords:** mesenchymal stem cells, replicative senescence, mitochondria, ROS

## Abstract

Mesenchymal stem cells (MSCs) are multipotent progenitors capable of self-renewal and differentiation into various cell lineages, making them essential for tissue repair and regenerative medicine. However, their regenerative potential is constrained by replicative senescence, an irreversible growth arrest that occurs after a finite number of cell divisions. In this study, we serially passaged human bone marrow-derived MSCs (bMSCs) and compared young, pre-senescent, and senescent cells. The onset of senescence was accompanied by progressive alterations in mitochondrial dynamics, leading to a decline in mitochondrial membrane potential, and increased reactive oxygen species (ROS) production, alongside a diminished cellular antioxidant capacity. These mitochondrial defects play a role in metabolic reprogramming in senescent bMSCs. Our findings underscore the intricate interplay between ROS, mitochondrial dysfunction, and replicative senescence, offering valuable insights to guide the development of therapeutic strategies for preserving MSC functionality in aging and MSC-based therapies.

## 1. Introduction

Cellular senescence is a state of irreversible growth arrest originally identified as a tumor-suppressive mechanism [1]. However, it is now widely recognized as a key contributor to aging and age-related diseases due to its involvement in chronic inflammation, tissue dysfunction, and diminished regenerative capacity [2,3]. Senescence also emerged as having a role in tissue remodeling during development and in wound healing [4]. Senescence occurs in response to various stressors, including DNA damage, oxidative stress, and oncogene activation [4], while replicative senescence, first described by Hayflick’s seminal work [5], ensues after a finite number of cell divisions due to progressive telomere shortening and is considered a hallmark of aging [3]. Senescent cells exhibit similar features, including structural alterations, telomere attrition, mitochondrial dysfunction, metabolic reprogramming, and disrupted intercellular communication [3]. Although these characteristics are broadly shared across senescent cell types, differences exist among various cell populations.

This study focuses on human mesenchymal stem cells (MSCs), which play an essential role in maintaining tissue homeostasis. Bone marrow-derived MSCs (bMSCs), in particular, are distinguished by their self-renewal capacity and multilineage differentiation potential [6]. These spindle-shaped cells are integral to the bone marrow niche, promoting bone formation and secreting soluble factors that regulate and sustain hematopoietic progenitors [7]. Like other somatic cells, bMSCs have a finite lifespan and undergo structural and functional changes with aging [8,9]. In animal models, biological aging of bMSCs leads to reduced cellular proliferation, impaired differentiation, increased oxidative stress, hindered DNA damage repair, and telomere shortening [10]. In vitro, two primary factors drive these changes [11]: the donor’s age at the time of biopsy and replicative senescence experienced during cell culture [5]. Morphological, molecular, and functional alterations have been reported in bMSCs isolated from young and old healthy donors, as well as in bMSCs subjected to serial passaging in vitro [12,13]. Aging and replicative senescence exert interconnected effects on human stem cells [14]. Replicatively senescent and aging bMSCs remain mitotically arrested but metabolically active, exhibiting similar gene expression profiles [15].

Given their regenerative potential, bMSCs have garnered considerable interest as therapeutic tools in tissue engineering and regenerative medicine [16,17]. Additionally, due to their ability to sense inflammatory conditions and suppress immune responses, they have been utilized in cell-based therapies for immune-mediated inflammatory diseases [18]. However, for in vivo applications, these cells must be expanded in vitro, a process that inevitably promotes replicative senescence. Understanding the molecular signatures and mechanisms underlying replicative senescence is crucial for gaining deeper insights into the senescence process and enhancing the reliability and effectiveness of MSC-based therapies. While previous studies have explored structural and functional changes in aging bMSCs, our study offers new insights into the gradual changes occurring when bMSC are passaged in vitro. Using human bMSCs at passages 4, 11, and 16, we sought to model the progressive stages of replicative senescence.

Our analysis focused on mitochondrial function and reactive oxygen species (ROS) generation, key factors implicated in cellular aging and senescence, alongside metabolic rewiring.

## 2. Materials and Methods

### 2.1. Culture of Human bMSCs

Human bone marrow-derived mesenchymal stem cells (bMSCs), purchased from CliniSciences (Nanterre, France), were obtained from a female healthy donor and cultured in Dulbecco’s Modified Eagle’s Medium (DMEM) supplemented with 20% fetal bovine serum (FBS), 2 mM glutamine, 10,000 U/mL penicillin, and 10 mg/mL streptomycin. After reaching confluence, the cells were trypsinized and counted using an automated cell counter (Logos Biosystems, Dongan-gu, Anyang-si, Republic of Korea). The cells were maintained at 37 °C in a humidified environment with 5% CO_2_. Replicative senescence was induced by serial passage [5]. To evaluate Senescence-Associated β-galactosidase (SA-β-gal) bMSCs were seeded on a 24-well plate (Costar, Sigma-Aldrich, St. Louis, MO, USA). Cells were then washed with phosphate-buffered saline (PBS), fixed in 2% formaldehyde and 0.2% glutaraldehyde for 10–15 min at room temperature, and subsequently incubated overnight at 37 °C with a freshly prepared staining solution containing 1 mg/mL X-Gal (B4252, Sigma-Aldrich), 40 mM citric acid/sodium phosphate buffer (pH 6.0), 5 mM potassium ferrocyanide, 5 mM potassium ferricyanide, 150 mM NaCl, and 2 mM MgCl_2_. The following day, cells were rinsed with PBS and examined under a bright-field microscope to detect blue-stained senescent cells. Images were captured at 10× magnification. The blue-colored precipitates were quantified at 650 nm using the Varioskan LUX Multimode Microplate Reader (Thermo Fisher Scientific, Waltham, MA, USA).

All reagents were from Sigma (Sigma-Aldrich) and sterile plastic was from Euroclone (Pero, Milan, Italy).

### 2.2. Western Blot

3 × 10^5^ cells were lysed in a buffer containing 50 mM TrisHCl (pH 7.4), 150 mM NaCl, 1% NP-40, and 0.25% sodium deoxycholate, supplemented with protease inhibitors (10 µg/mL Leupeptin, 10 µg/mL Aprotinin, 1 mM PMSF). Protein concentration was determined using Bradford assay (B6916, Sigma-Aldrich), and 20–40 µg of total protein per lane were separated on SDS-PAGE and transferred to nitrocellulose membranes using the Trans-Blot Turbo Transfer System (1704158, Bio-Rad, Hercules, CA, USA). After blocking with 5% bovine serum albumin (BSA), Western blots were performed using primary antibodies (diluted in 5% BSA) targeting p21, Optic Atrophy 1 (OPA1), Dynamin-Related Protein 1 (Drp1) (2947S, 80471S, 5391S, Cell Signaling Technology, Danvers, MA, USA), BCL2 interacting protein 3 (BNIP3), Cyclophilin F (CYP F) (MA1-24688, E11AE12BD4, Invitrogen, Thermo Fisher Scientific), lipid droplet protein perilipin 2 (PLIN2) (AB108323, Abcam, Cambridge, UK), with glyceraldehyde 3-phosphate dehydrogenase (GAPDH) (sc-25778, Santa Cruz Biotechnology, Dallas, TX, USA) serving as a loading control. After extensive washing in Tris-buffered saline with 0.1% Tween, membranes were incubated with horseradish peroxidase-conjugated secondary antibodies (Amersham Pharmacia Biotech, GE Healthcare, Heller International Building, Chicago, IL, USA), and bands were visualized using Clarity™ Western ECL substrate (170-5061, Bio-Rad). Signal detection was carried out using the ChemiDoc MP Imaging System (Bio-Rad), and densitometric analysis was performed with ImageLab software (Java 1.8.0_241, Bio-Rad). A representative blot is shown.

### 2.3. Confocal Microscopy

bMSCs cultured on microscope slides (631-0149, VWR International S.r.l., Radnor, PA, USA) (3 × 10^4^ cells/well) were fixed in PBS containing 4% paraformaldehyde and 2% sucrose (pH 7.6) for 15 min. Permeabilization and blocking were performed for 30 min using a PBS solution containing 2% BSA and 0.3% Triton. Cells were incubated overnight at 4 °C with antibodies anti-CYP F (Invitrogen, Thermo Fisher Scientific), then labeled with Alexa Fluor 488 secondary antibodies (A11001, Thermo Fisher Scientific, Waltham, MA, USA). Cytoskeleton was visualized using TRITC-labeled phalloidin (R415, Thermo Fisher Scientific) and nuclei were stained with 4’,6-diamidino-2-phenylindole (DAPI) (62248, Sigma-Aldrich). Samples were mounted using Mowiol, and images were captured using a Leica SP8 confocal microscope (Leica Microsystems, Wetzlar, Germany) with a 40× oil immersion objective.

The major radius of the nuclei was measured using the LAS X Life Science Microscope Software (3.7.1.21655, Leica Microsystems). The data are expressed as a box plot graph.

Mitochondria were analyzed using the MiNA plugin of the ImageLab software (Bio-Rad). The branch length mean, summed branch length mean, and network branches mean are presented as a box plot graph.

### 2.4. Real-Time PCR (RT-PCR)

Total RNA was isolated from bMSCs (4 × 10^4^ cells/well) using the PureLink RNA Mini Kit (12183018A, Invitrogen, Thermo Fisher Scientific) following the manufacturer’s instructions. cDNA was synthesized in a final reaction volume of 20 µL using the High-Capacity cDNA Reverse Transcription Kit with RNase Inhibitor (4368814, Applied Biosystems, Thermo Fisher Scientific). Quantitative RT-PCR was performed on the CFX96 Real-Time PCR Detection System (Bio-Rad) using TaqMan Gene Expression Assays (Life Technologies, Monza, Italy) for target genes: Cyclin Dependent Kinase Inhibitor 1A (*CDKN1A*) (Hs003555782_m1) which encodes p21 and mitochondrially encoded NADH dehydrogenase-1 (*MT-ND1*) (Hs02596873_s1), with *GAPDH* (Hs99999905_m1) serving as the internal reference gene. Gene expression levels were determined using the 2^−ΔΔCt^ method.

### 2.5. Protein Array

Conditioned media from bMSCs (1.5 × 10^6^) were collected after 3 days of culture. After centrifugation, the media were used to incubate the membrane on which 40 antibodies against proteins involved in inflammation were spotted in duplicate (ARY005B, RayBiotech, Norcross, GA, USA) as described [19]. The densitometric analysis of each spot of the array was analyzed with ImageLab software (Bio-Rad).

### 2.6. MitoSOX

In spite of some limitations [20], MitoSOX is widely utilized to quantify mitochondrial ROS [21]. bMSCs were cultured in a 96-well black plate (Costar, Sigma-Aldrich) (6 × 10^3^ cells/well) and incubated for 10 min at 37 °C with MitoSOX (M36008, Molecular Probes, Thermo Fisher Scientific), protected from light. Fluorescence was measured at λex/λem = 510/580 nm using the Varioskan LUX Multimode Microplate Reader (Thermo Fisher Scientific), and normalized to the cell number.

### 2.7. Reduced/Oxidized Glutathione

The ratio of reduced (GSH)/oxidized (GSSG) glutathione was evaluated using the luminescence-based GSH/GSSG-Glo Assay (Promega, Madison, WI, USA). bMSCs seeded on a 96-well white plate (Costar, Sigma-Aldrich) (6 × 10^3^ cells/well) were incubated with Total Glutathione Lysis Reagent or Oxidized Glutathione Lysis Reagent and shaken at room temperature for 5 min. Then, Luciferin Generation Reagent was added to each well and the cells were incubated at room temperature for 30 min. Luciferin Detection Reagent was then added to each well. After 15 min, luminescence was acquired using Varioskan LUX Multimode Microplate Reader (Thermo Fisher Scientific).

### 2.8. Mitochondrial Membrane Potential (ΔΨm)

Changes in mitochondrial membrane potential were analyzed by staining bMSCs with 10 µg/mL JC-1 (T3168, Invitrogen, Thermo Fisher Scientific). This probe evaluates mitochondrial potential by measuring fluorescence intensities of red-shifted aggregates (in functional mitochondria) and green-shifted JC-1 (in damaged mitochondria) monomers. Cells seeded on a 96-well black plate (Costar, Sigma-Aldrich) (6 × 10^3^ cells/well) were incubated for 15 min at 37 °C in the dark with JC-1 (λex/λem red = 535/590 nm; λex/λem green = 485/530 nm). Fluorescence was acquired at Varioskan LUX Multimode Microplate Reader (Thermo Fisher Scientific). The red/green ratio was calculated for each sample. Mitochondrial membrane depolarization was assessed by changes in the JC-1 red/green fluorescence ratio, with a decreased ratio indicating a reduction in mitochondrial membrane potential [22]. Cells cultured on microscope slides (631-0149, VWR International S.r.l.) and incubated with JC-1 were also imaged using a 40× oil-immersion objective on an Leica SP8 confocal microscope (Leica Microsystems).

### 2.9. Fatty Acid Oxidation (FAO) Analysis

Fatty Acid Oxidation Assay (ab222944, Abcam) was used to monitor FAO, the primary metabolic pathway for degradation of fatty acids, in living cells. bMSCs seeded on 96-well black plate (Costar, Sigma-Aldrich) (6 × 10^3^ cells/well) were rinsed twice with Fatty Acid-Free medium added with 0.5 mM L-carnitine and 2.5 mM D-Glucose. In the meantime, Fatty Acid Measurement Medium was prepared by adding Oleate (FAO-conjugate) to Fatty Acid-Free Medium and added to each assay well. Extracellular O_2_ Consumption Reagent (λex/λem = 380/650 nm) was then added to the wells, and each well was sealed with pre-warmed High Sensitivity Mineral Oil. Treatment with the mitochondrial membrane potential uncoupler carbonyl cyanide-p-trifluoromethoxyphenylhydrazone (FCCP, 0.625 µM) was used as positive control. The FAO inhibitor Etomoxir (40 μM) was utilized as a negative control Fluorescence was acquired every 1.30 min for 90 min at Varioskan LUX Multimode Microplate Reader pre-set to 37 °C. Data were normalized to the cell number. The results are expressed as a box plot graph.

### 2.10. ATP Quantification

ATP levels were quantified using the CellTiter-Glo Luminescent Cell Viability Assay (G7571, Promega). This assay is based on a thermostable luciferase that, in the presence of Mg^2+^, catalyzes an oxidative reaction, generating bioluminescence. Specifically, luciferin, along with molecular oxygen and ATP as cofactors, is converted into oxyluciferin, resulting in light emissions. bMSCs seeded on a 96-well white plate (Costar, Sigma-Aldrich) (6 × 10^3^ cells/well) were incubated with CellTiter-Glo Reagent, diluted in culture medium with a 1:1 ratio, for 10 min at room temperature. The luciferase activity was monitored using the Varioskan LUX Multimode Microplate Reader (Thermo Fisher Scientific). Data were normalized to the cell number.

### 2.11. Triglyceride (TG) Quantification

The Triglyceride Quantification Kit (MAK266-1KT, Sigma-Aldrich) was used to quantify TGs in bMSCs. The cells were seeded in a 6-well plate (Costar, Sigma-Aldrich) (1.5 × 10^5^ cells/well). After 3 days, the cells were lysed in 1 mL of H_2_O + 5% NP-40 and centrifuged for 2 min at 13,000 rpm. Cells (6 × 10^3^) were plated in triplicate in a 96-well black plate (Costar, Sigma-Aldrich). Lipase was then added to each well for 20 min at room temperature. The glycerol generated from the hydrolysis of TGs was then oxidized by adding the Master Reaction Mix provided in the kit, resulting in the generation of a fluorescent product (λex/λem = 535/587 nm). The fluorescence was acquired at Varioskan LUX Multimode Microplate Reader (Thermo Fisher Scientific). A standard curve was generated to calculate the TG concentration in each sample (ng/μL).

### 2.12. Lipid Droplet Staining

bMSCs seeded on 96-well black plate (Costar, Sigma-Aldrich) (6 × 10^3^ cells/well) were incubated for 40 min at 37 °C in the dark with Bodipy™ (λex/λem = 493/503 nm) and Hoechst 33342 (λex/λem = 361/497 nm) (D3922, 62249, Thermo Fisher Scientific) [23]. Fluorescence was acquired at Varioskan LUX Multimode Microplate Reader and normalized to the cell number. For images acquisition, bMSCs were cultured on 24-well plates, stained with Bodipy and Hoechst, and fixed in 2% formaldehyde and 0.2% glutaraldehyde for 15 min at room temperature. Images were acquired using FLoid™ Cell Imaging Station (Thermo Fisher Scientific).

### 2.13. Intracellular Lactate Quantification

The luminescence-based Lactate-Glo™ Assay (J5021, Promega) was used to quantify intracellular lactate. Specifically, lactate dehydrogenase uses lactate and NAD^+^ to produce pyruvate and NADH. In the presence of NADH, a pro-luciferin reductase substrate is converted by reductase into luciferin, which is subsequently used in a luciferase reaction to generate light. Therefore, the light emission is proportional to the amount of lactate in each sample. bMSCs were seeded in a 6-well plate (Costar, Sigma-Aldrich) (1.5 × 10^5^ cells/well) and, after 3 days, were detached from the plate and counted for normalization. Cells were then incubated for 5 min at room temperature with Inactivation Solution (0.6 N HCl in H_2_O) which rapidly stops metabolism, lyses the cells, inhibits activity of endogenous proteins and destroys reduced NAD(P)H dinucleotides. After inactivation, Neutralization Solution (1 M Trizma^®^) was added to each sample. The samples were aliquoted in duplicate into a 96-well white plate (Costar, Sigma-Aldrich). At the same time, Lactate Detection Reagent was prepared according to the manufacturer’s instructions and added to each well. The plate was incubated for 1 h at room temperature in the dark. Luminescence was measured using a Varioskan LUX Multimode Microplate Reader (Thermo Fisher Scientific). The results were normalized to the cell number.

### 2.14. Statistical Analysis

All experiments were performed in triplicate and independently repeated at least three times. Data are presented as mean ± standard deviation (SD). The data are analyzed with GraphPad Prism 6.01. The data were normally distributed and analyzed using one-way repeated measures ANOVA. The *p*-values deriving from multiple pairwise comparisons were corrected by the Bonferroni method. Statistical significance was defined for *p*-value ≤ 0.05. * *p* ≤ 0.05; ** *p* ≤ 0.01; *** *p* ≤ 0.001.

## 3. Results

### 3.1. Human bMSCs Exhibit Scenescence Markers at Passages 11 and 16

**Figure 1:**
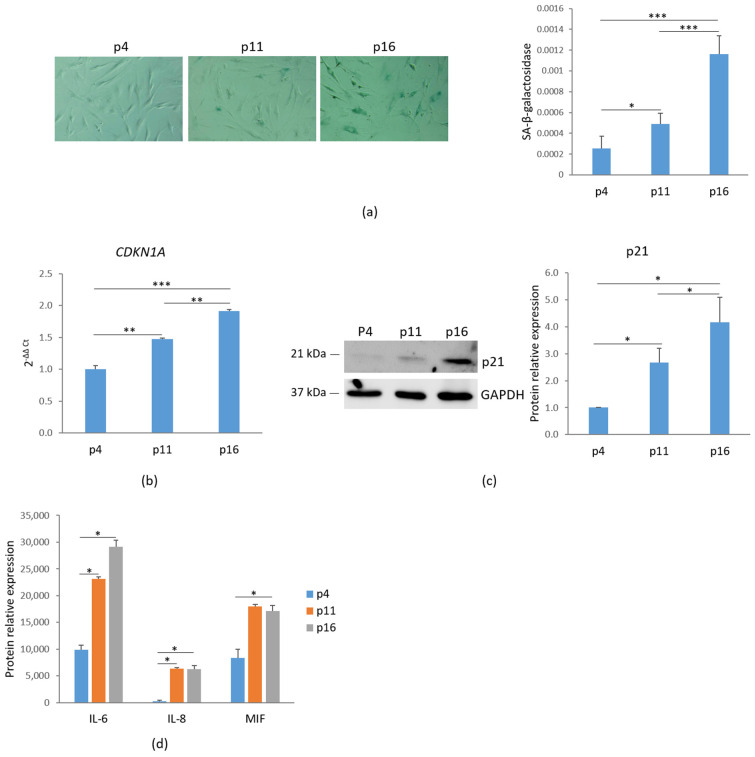
Characterization of senescent markers in bMSCs. (**a**) bMSCs at passage (p) 4, 11, and 16 were analyzed for β-galactosidase. The photos were taken using a 10× objective on an optical microscope (**left panel**) and the blue-colored precipitates were quantified at 650 nm (**right panel**). (**b**) *CDKN1A* was analyzed by RT-PCR. (**c**) p21 levels were assessed by Western blot with GAPDH used as a loading control. A representative blot (**left panel**) and densitometry (**right panel**) performed on three independent experiments using ImageLab are shown. (**d**) The levels of some senescence-associated cytokines in the media were analyzed by protein array. * *p* ≤ 0.05; ** *p* ≤ 0.01; and *** *p* ≤ 0.001.

To assess cellular senescence, bMSCs at various passages were analyzed for SA-β-gal activity, a widely used marker of senescence. Using 5-bromo-4-chloro-3-indolyl-β-D-galactopyranoside (X-Gal) as a substrate, β-galactosidase catalyzes its conversion into galactose and 5-bromo-4-chloro-3-hydroxyindole, which dimerizes to form blue-colored precipitates. As shown in Figure 1a, left panel, blue precipitates were detected in passage (p) 11 bMSCs, with a significant increase in p16 cells, while no detectable staining was observed in p4 bMSCs. The absorbance measurement of the blue aggregates indicates an increase in SA-β-gal staining (Figure 1a, right panel). Another key marker of senescence, the nuclear protein p21, encoded by the *CDKN1A* gene and responsible for governing cell cycle arrest, was analyzed. A significant, gradual increase in *CDKN1A* RNA and p21 levels was observed in bMSCs as they progressed through serial passaging in vitro (Figure 1b,c). Based on these data, hereafter, we refer to bMSCs at passage 4 as ‘young’, those at passage 11 as ‘pre-senescent’, and those at passage 16 as ‘senescent’.

Senescence-associated secretory phenotype (SASP) is another feature of cellular senescence [3]. We evaluated the levels of some cytokines in the media of bMSCs at different passages and found a marked increase in the levels of interleukin (IL)-6, IL-8 and Macrophage Migration Inhibitory Factor (MIF) (Figure 1d).

### 3.2. Pre-Senescent and Senescent bMSCs Exhibit Morphological and Mitochondrial Alterations

**Figure 2:**
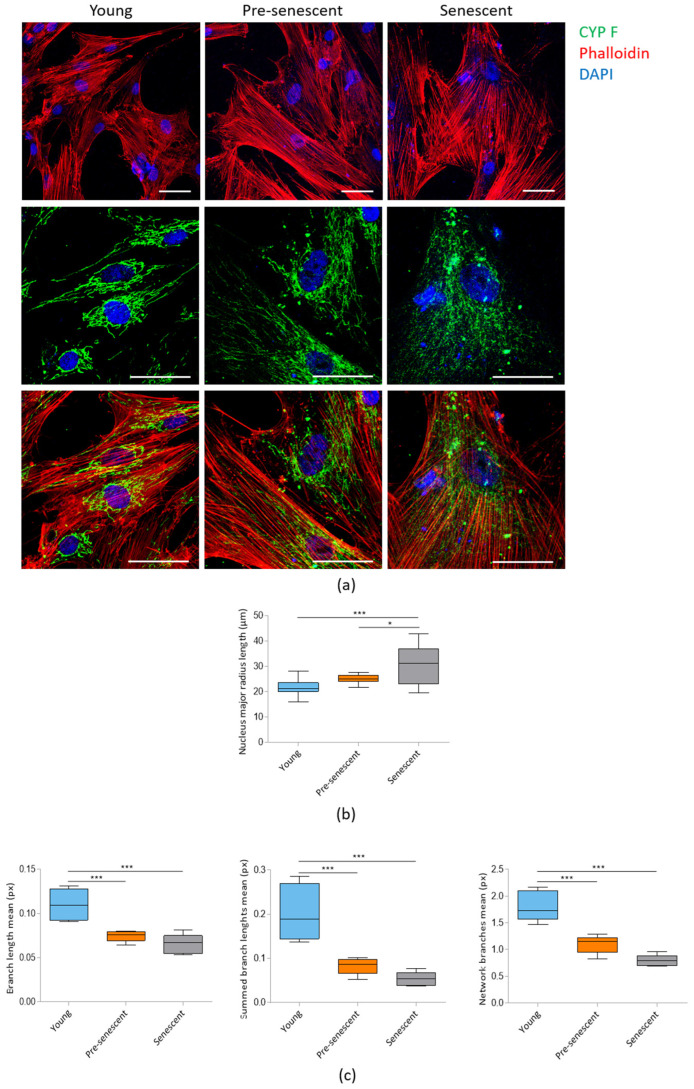
Confocal imaging of bMSCs at various passages. (**a**) Young, pre-senescent and senescent bMSCs were analyzed by confocal microscopy after CYP F (green), phalloidin (red) and DAPI (blue) staining. Scale bar: 50 µm. The major radius of the nuclei (**b**) and the branch length mean, summed branch length mean, and network branches mean of mitochondria (**c**) were analyzed in young, pre-senescent, and senescent bMSCs. * *p* ≤ 0.05; *** *p* ≤ 0.001.

Confocal microscopy following actin staining with phalloidin revealed a significant increase in cell size in pre-senescent and senescent bMSCs compared to young cells (Figure 2a, upper panel). The increase in cell size is associated with enlarged nuclei, as detected by confocal images (Figure 2a) and demonstrated by analyzing the major nuclear radius length (Figure 2b). Numerous prominent actin fibers traversing the cytoplasm could be detected in all cells, indicating that senescent bMSCs maintain cytoskeletal organization. Mitochondrial morphology was assessed using antibodies against CYP F, a widely used tool for imaging mitochondria. Young cells exhibited an intricate mitochondrial network with elongated mitochondria surrounding the nucleus, whereas pre-senescent and senescent bMSCs showed fragmented, punctate mitochondria scattered throughout the cytoplasm (Figure 2a, middle and lower panels). The significant reduction in mitochondrial branch lengths in pre-senescent and senescent bMSCs was also calculated using MiNA plugin of the ImageLab software (Figure 2c).

**Figure 3:**
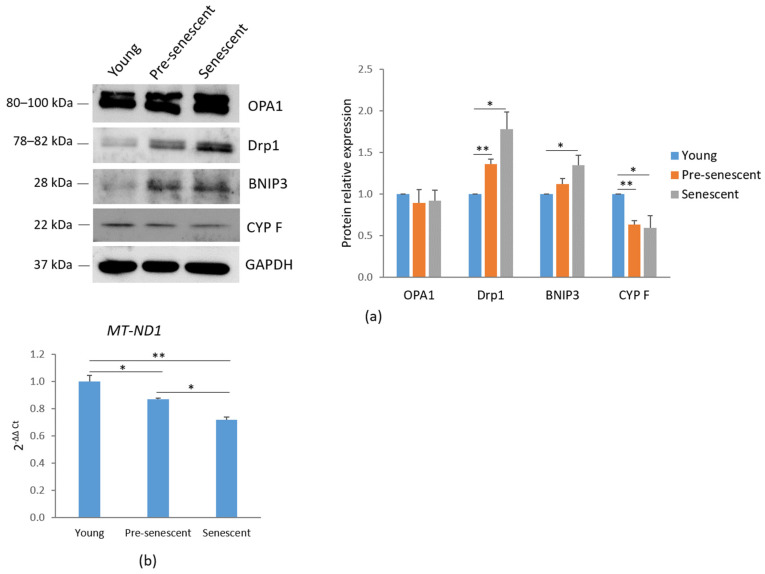
Mitochondrial dynamics and mitochondrial content in bMSCs at various passages. (**a**) Young, pre-senescent, and senescent bMSCs were analyzed by Western blot to evaluate the total amounts of OPA1, Drp1, BNIP3, CYP F. Anti-GAPDH antibodies were used as a control of equal loading. A representative blot (**left panel**) and densitometry (**right panel**) performed on three independent experiments using ImageLab are shown. (**b**) The mitochondrial content was analyzed by RT-PCR using primers specific to *MT-ND1*. * *p* ≤ 0.05; ** *p* ≤ 0.01.

Mitochondrial dynamics are regulated by several mechanisms, including biogenesis, mitophagy, fusion, and fission. A western blot analysis of mitochondria-shaping proteins revealed that the total amounts of OPA1, which regulates mitochondrial fusion [24], remained unchanged. However, a significant increase in Drp1, which mediates mitochondrial fission [25], was observed in pre-senescent and senescent bMSCs compared to young cells (Figure 3a). These findings correlate with the mitochondrial fragmentation observed in cells at increasing passage numbers.

Additionally, an increase in BNIP3, a receptor involved in mitochondrial autophagy (mitophagy) through direct interaction with LC3, was demonstrated in pre-senescent and senescent cells [26] (Figure 3a). In agreement with these findings, a reduction in CYP F levels was detected in pre-senescent and senescent bMSCs compared to young cells (Figure 3a), indicating a decline in mitochondrial content with serial passage in vitro. These data were also confirmed by RT-PCR using primers specific for the mtDNA gene *MT-ND1*, commonly used for mitochondrial DNA quantification due to its relatively stable copy number across different cell types and conditions (Figure 3b).

### 3.3. Pre-Senescent and Senescent bMSCs Show a Reduction in Mitochondrial Membrane Potential, an Increase in ROS Production and a Decrease in GSH/GSSG Ratio

**Figure 4:**
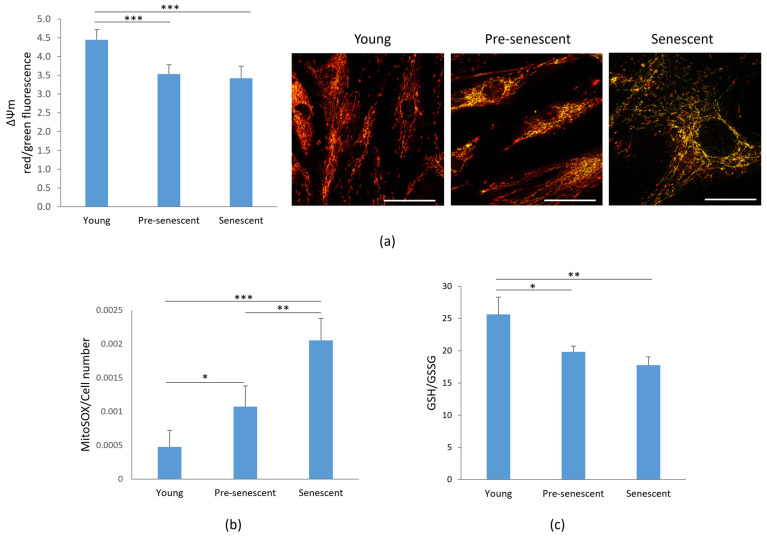
Mitochondrial membrane potential, ROS production and GSH/GSSG in bMSCs at various passages. Young, pre-senescent and senescent bMSCs were analyzed for (**a**) Mitochondrial membrane potential. After JC-1 staining the red/green fluorescence ratio was measured (**left panel**). A decreased ratio indicates a decreased mitochondrial membrane potential. In parallel, cells seeded on coverslips were stained with JC1 and visualized using a confocal microscopy (**right panel**); (**b**) Mitochondrial ROS production using the MitoSOX probe. The data were normalized to the cell number; (**c**) GSH/GSSG ratio which was evaluated using a luminescence-based assay. * *p* ≤ 0.05; ** *p* ≤ 0.01 and *** *p* ≤ 0.001.

Given the observed alterations in mitochondrial morphology, we next analyzed mitochondrial function. One key indicator of mitochondrial dysfunction is the alteration of membrane potential (ΔΨm) [27], which reflects the mitochondrion’s ability to generate ATP through oxidative phosphorylation [28]. Mitochondrial membrane potential was assessed by the JC-1 dye. A significant depolarization of the mitochondrial membrane potential, monitored by changes in the JC-1 red/green fluorescence ratio, was observed in pre-senescent and senescent bMSCs compared to young cells (Figure 4a, left panel). In parallel, the cells were cultured on microscope slides, incubated with JC-1, and observed using a confocal microscope (Figure 4a, right panel). Red-shifted aggregates identify functional mitochondria, while green-shifted JC-1 highlights damaged mitochondria.

Senescence is often associated with increased ROS production. Under physiological conditions, 1–4% of oxygen is reduced in mitochondria via a one-electron reduction, resulting in the formation of ROS, primarily superoxide anion (O_2_^−^) [29]. Using the fluorescent probe MitoSOX, we observed a progressive increase in mitochondrial ROS production in bMSCs as they underwent senescence in vitro (Figure 4b). Since GSH is the most abundant antioxidant in aerobic cells [30], we measured the GSH/GSSG ratio and observed a significant decrease in pre-senescent and senescent cells (Figure 4c).

### 3.4. Fatty Acid Oxidation (FAO) Is Reduced and Intracellular Neutral Lipids Are Increased in Pre-Senescent and Senescent bMSCs

**Figure 5:**
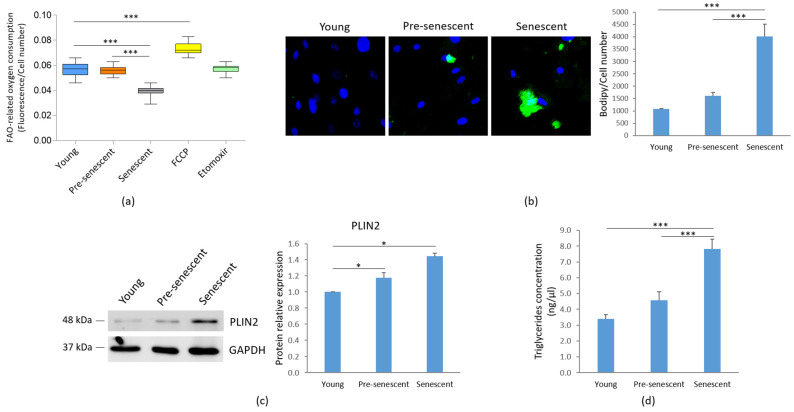
FAO, lipid droplet accumulation and TG content in bMSCs at various passages. Young, pre-senescent and senescent bMSCs were analyzed for (**a**) FAO, which was measured using the Fatty Acid Oxidation assay kit, as described in the Materials and Methods section. The FAO activator FCCP (0.625 μM) served as the positive control, while the FAO inhibitor Etomoxir (40 μM) was used as the negative control; (**b**) Intracellular lipid droplet content after staining with the neutral fluorescent probe Bodipy^©^. Nuclei were stained with Hoechst 33342. Images were acquired using a fluorescence microscope (10× magnification, **left panel**). Fluorescence was acquired at Varioskan LUX Multimode Microplate Reader (**right panel**); (**c**) PLIN2 levels by Western blot performed on the same filter used in Figure 1c. GAPDH was used as control of loading. A representative blot (**left panel**) and densitometry performed on three independent experiments using ImageLab (**right panel**) are shown; (**d**) TG content, which was measured using the Triglyceride Quantification Kit. * *p* ≤ 0.05; *** *p* ≤ 0.001.

We analyzed FAO as an indicator of mitochondrial metabolic activity. A significant decline of FAO was observed in senescent bMSCs compared to younger cells (Figure 5a).

Concomitantly, as indicated by Bodipy© staining for intracellular neutral lipids, an accumulation of lipid droplets was observed in pre-senescent and senescent bMSCs, although statistical significance was reached only in senescent cells (Figure 5b). The increase in lipid droplet content in both pre-senescent and senescent cells was also confirmed by the upregulation of PLIN2, a widely used protein marker for lipid droplets [31] (Figure 5c). Moreover, a significant increase in TG levels was detected in senescent bMSCs compared to young cells (Figure 5d).

### 3.5. Lactate Production Is Increased, While ATP Levels Are Reduced in Senescent bMSCs

**Figure 6:**
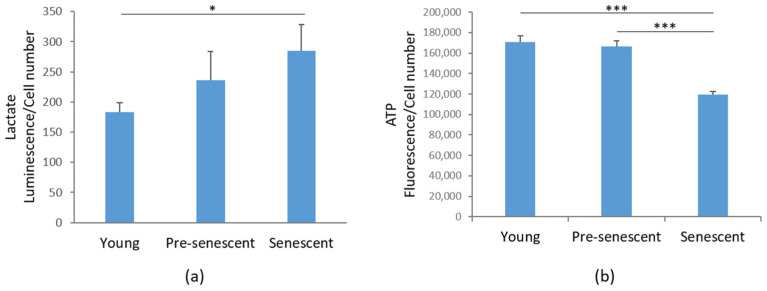
Lactate and ATP production in bMSCs at various passages. (**a**) Lactate production was measured in young, pre-senescent, and senescent bMSCs and normalized to the cell number. (**b**) ATP levels were quantified using the CellTiter-Glo Luminescent Cell Viability Assay. The results were normalized to the cell number. * *p* ≤ 0.05; *** *p* ≤ 0.001.

Then, we investigated glycolytic metabolism by measuring the amounts of lactate, the end product of glycolysis [32]. Interestingly, in senescent bMSCs, a significant increase in intracellular lactate content was demonstrated compared to young bMSCs (Figure 6a). An increase in lactate was also detected in pre-senescent cells, even though it did not reach statistical significance (Figure 6a). Nevertheless, ATP production was significantly reduced in senescent cells (Figure 6b).

## 4. Discussion

bMSCs possess key characteristics that make them a promising source for cell therapy, including their multipotency and ease of isolation from various sources. They can be rapidly expanded on a large scale for clinical applications and are advantageous in diverse therapeutic contexts due to their ability to home to injured tissues via chemoattraction. However, emerging evidence indicates that replicative senescence markedly diminishes their therapeutic efficacy [33]. Consequently, a deeper understanding of replicative senescence during in vitro culture is essential for advancing stem cell-based therapies. Our study provides a characterization of the progressive stages of replicative senescence in human bMSCs, with a particular focus on mitochondrial function, ROS generation and metabolic rewiring.

Initially, we demonstrate that as bMSCs age in vitro, they exhibit morphological alterations, increased SA-β-gal activity, upregulated p21, and the development of a senescence-associated secretory phenotype (SASP), all of which are well-established markers of cellular senescence [34]. SA-β-gal, a lysosomal hydrolase, shows detectable activity in senescent cells under suboptimal pH conditions, primarily due to an increase in lysosomal number and size [35]. p21 is a strong, tight-binding inhibitor of all cyclin–cyclin-dependent kinase complexes, leading to cell cycle arrest [36]. Furthermore, pre-senescent and senescent bMSCs released higher amounts of IL-6 and IL-8 than young cells. These cytokines play a crucial role in reinforcing senescence-induced growth arrest through both autocrine and paracrine signaling mechanisms [37,38]. Therefore, the mechanisms regulating IL-6 and IL-8 expression in senescent cells are of significant interest and may hold key implications for developing strategies to mitigate cellular senescence. Also, the secretion of MIF, which has been implicated not only in inflammation but also in immune evasion, progressively increased as bMSCs age in vitro. Interestingly, MIF was overexpressed in bones from old vs. young mice and was significantly reduced following the genetic clearance of senescent cells in the old mice [39]. As cell enlargement has long been recognized as a biomarker of senescence both in vitro [2] and in vivo [40], it is noteworthy that bMSCs gradually enlarge with serial passage, likely due to the fact that senescent cells accumulate biomass without dividing. Maintaining a specific cell size is crucial for normal cellular function. Recent findings suggest that increased cell size is not merely a consequence but also a driver of permanent cell cycle exit, as it disrupts normal cellular physiology and induces homeostatic imbalances that contribute to senescence [41]. This is an intriguing point of view that deserves further investigation in senescent bMSCs. Confocal microscopy also highlighted relevant alteration of mitochondria. In young bMSCs an intricate mitochondrial network characterized by elongated perinuclear mitochondria was observed, whereas pre-senescent and senescent bMSCs showed fragmented mitochondria scattered in the cytosol. These mitochondrial alterations suggest an imbalance between fission and fusion in senescent cells. Fission is a multi-step process that separates damaged mitochondria from their healthy counterparts and is followed by selective removal of damaged mitochondria through mitophagy [25]. Of note, with the increase in passages in vitro, bMSCs accumulate Drp1, a mitochondrial fission regulator crucial for controlling mitochondrial integrity and metabolic homeostasis [25]. Also, BNIP3, a known mitochondrial quality regulator [42], was upregulated in senescent bMSCs. In addition to its critical role in mitophagy, BNIP3 also promotes mitochondrial fission through its interaction with OPA1, a dynamin of the mitochondrial inner membrane that regulates fusion [43]. The BNIP3-OPA1 interaction might thus enhance Drp1-driven fission by inhibiting the antagonistic fusion activity of OPA1. We propose that BNIP3 upregulation might play a central role in altering mitochondrial dynamics in senescent bMSCs by reducing fusion and promoting fission. Further functional experiments would be needed to establish a causal relationship linking BNIP3 to mitochondrial fission and fusion in senescent bMSCs.

Contrary to previous reports suggesting an increase in mitochondrial mass during senescence both in vitro [27] and in vivo [27], our findings indicate a reduction in mitochondrial content in senescent bMSCs, as evidenced by decreased CYP F levels and *MT-ND1* copy number, likely driven by BNIP3 upregulation. Despite the elevated BNIP3 expression, which is typically linked to enhanced mitophagy, it is likely that not all dysfunctional mitochondria are effectively removed. Indeed, in pre-senescent and senescent bMSCs, we observed a decline in mitochondrial membrane potential.

ROS accumulation is a well-established driver of cellular senescence and aging-related pathologies [1]. We observed a significant increase in ROS production, as detected by the MitoSOX probe. The overproduction of ROS leads to oxidative damage in macromolecules, further promoting cellular dysfunction and also fueling the SASP [44]. Consistent with previous reports, our data suggest that oxidative stress is both a cause and consequence of mitochondrial dysfunction in senescent bMSCs. We also report a reduced GSH/GSSG ratio as senescence progresses, indicating a senescence-related decline in the cellular antioxidant defense system. Boosting glutathione levels-via N-acetylcysteine (NAC), dietary precursors, or metabolic interventions-may be a potential strategy to slow replicative senescence, aging and combat age-related diseases.

Metabolic reprogramming is emerging as a hallmark of cellular senescence. A key finding of our study is the reduction in FAO activities and ATP content, alongside the accumulation of lactate in senescent bMSCs. Elevated lactate levels, the end product of glycolysis, can further impair FAO, as lactate competes with fatty acids for mitochondrial oxidation [45]. Impaired FAO has been implicated in the decline of bMSC functionality [46], further reinforcing the importance of mitochondrial homeostasis in maintaining stem cell regenerative capacity. In parallel, senescent bMSCs exhibit an abnormal accumulation of TGs, along with an increase in intracellular lipid droplets, which function as reservoirs for excess fat. The increase in lipid droplets can be interpreted as an adaptive mechanism to safeguard senescent bMSCs from lipotoxicity [47]. It is worth noting that morphological and mitochondrial alterations develop gradually, whereas differences in lipid metabo-lism, lactate synthesis, and ATP production reach statistical significance only in senescent cells. We hypothesize that there is a threshold of mitochondrial impairment beyond which significant metabolic differences become evident.

The progressive decline in mitochondrial function observed in our study has significant implications for bMSC-based regenerative therapies. Oxidative stress, metabolic reprogramming, and pro-inflammatory signaling impair the regenerative functions of bMSCs. Targeting mitochondrial pathways may offer promising strategies to improve bMSC longevity and function in regenerative medicine.

We acknowledge that our experiments could benefit from additional tools-such as the extracellular flux assay (Seahorse assay), as well as proteomic and transcriptomic approaches-to enhance our understanding on metabolic alterations and provide an integrated view of the complexity of senescence in cells with important therapeutic applications. We have now included these approaches as potential avenues for future studies. Additionally, we recognize that incorporating more time points would allow for a more detailed characterization of the gradual onset of senescence. A limitation of this study is that the reported findings are based on primary cells derived from a single donor, which may not fully capture the broader biological variability.

## 5. Conclusions

Our findings collectively highlight a progressive deterioration in structural integrity, mitochondrial function, and metabolic homeostasis, accompanied by increased oxidative stress in bMSCs undergoing replicative senescence. Investigating the interplay between mitochondrial dynamics, ROS generation and cellular metabolism may provide deeper insights into the mechanisms governing bMSC replicative senescence. Future research should explore interventions that mitigate mitochondrial impairment and oxidative stress to optimize MSC-based therapies for clinical applications.

## Data Availability

The data presented in this study are openly available in Dataverse through the following link: https://dataverse.unimi.it/dataverse/cellularsenescence (accessed on 12 February 2025).

## Abbreviations

The following abbreviations are used in this manuscript:

bMSCs	Bone Marrow Mesenchymal Stem Cells
ROS	Reactive Oxygen Species
DMEM	Dulbecco’s Modified Eagle’s Medium
FBS	Fetal Bovine Serum
SA-β-gal	Senescence-Associated β-galactosidase
PBS	Phosphate-Buffered Saline
DAPI	4′,6-diamidino-2-phenylindole
OPA1	Optic Atrophy 1
BSA	Bovine Serum Albumin
Drp1	Dynamin-Related Protein 1
BNIP3	BCL2 Interacting Protein 3
CYP F	Cyclophilin F
PLIN2	Lipid droplet protein perilipin 2
GAPDH	Glyceraldehyde 3-Phosphate Dehydrogenase
BSA	Bovine Serum Albumin
CDKN1A	Cyclin Dependent Kinase Inhibitor 1A
MT-ND1	Mitochondrially encoded NADH Dehydrogenase-1
GSH	Glutathione
GSSG	Glutathione Disulfide
FAO	Fatty Acid Oxidation
TG	Triglyceride
X-Gal	5-bromo-4-chloro-3-indolyl-β-D-galactopyranoside
SASP	Senescence-Associated Secretory Phenotype
IL	Interleukin
MIF	Macrophage Migration Inhibitory Factor
